# Hyperactive delirium during emergency department stay: analysis of risk factors and association with short-term outcomes

**DOI:** 10.1007/s11739-023-03440-3

**Published:** 2023-10-21

**Authors:** Òscar Miró, Gina Osorio, Aitor Alquézar-Arbé, Sira Aguiló, Cesáreo Fernández, Guillermo Burillo, Javier Jacob, Pere Llorens, Lluís Llauger, Ángel Peláez González, Edmundo Ramón Figuera Castro, Ricardo Juarez González, María José Blanco Hoffman, Fátima Fernandez Salgado, Teresa Pablos Pizarro, María Amparo Berenguer Díez, Marina Truyol Más, Nieves López-Laguna, Jacinto Garcia Acosta, Carmen Fernandez Domato, Francisco Javier Diego Robledo, Patxi Ezponda, Andrea Martinez Lorenzo, Juan Vicente Ortega Liarte, Inmaculada García Rupérez, Setefilla Borne Jerez, Claudia Corugedo Ovies, Blanca Andrea Gallardo Sánchez, Juan González Del Castillo, the researchers of the SIESTA network, Juan González Del Castillo, Cesáreo Fernandez Alonso, Jorge Garcia Lamberechts, Ana Chacon García, Paula Queizan García, Andrea Peirago Bravo, Alejandro Melcon Villalibre, Sara Vargas Lobé, Cristina Güemes de la Iglesia, Beatriz Honrado Galán, Sandra Moreno Ruíz, Osvaldo Jorge Troiano Ungerer, Enrique Martin Mojarro, Guillermo Burillo Putze, Aarati Vaswani Bulchand, Patricia Eiroa Hernández, Patricia Parra Esquivel, Pascual Piñera Salmerón, Inés García Rosa, María Consuelo Quesada Martínez, Marta Isabel Gómez Gómez, Yurena Reverte Pagán, Patricia Gantes Nieto, Virginia Arroyo Linares, Sara Rodrigo González, Sònia Jiménez, Sira Aguiló Mir, Francesc Xavier Alemany González, María Florencia Poblete Palacios, Claudia Lorena Amarilla Molinas, Gina Osorio, Sandra Cuerpo Cardeñosa, Matilde González Tejera, Ana Puche Alcaraz, Cristina Chacón García, Leticia Serrano Lázaro, Javier Millán Soria, Jésica Mansilla Collado, María Bóveda García, Pere Llorens Soriano, Adriana Gil Rodrigo, Begoña Espinosa Fernández, Mónica Veguillas Benito, Sergio Guzmán Martínez, Gema Jara Torres, María Caballero Martínez, Javier Jacob Rodríguez, Ferran Llopis, Elena Fuentes, Lidia Fuentes, Francisco Chamorro, Lara Guillen, Nieves López, Coral Suero Méndez, Lucía Zambrano Serrano, Rocío Muñoz Martos, Manuel Salido Mota, Valle Toro Gallardo, Antonio Real López, Lucía Ocaña Martínez, Esther Muñoz Soler, Mario Lozano Sánchez, Eva Fraguero Blesa, Fahd Beddar Chaib, Rodrigo Javier Gil Hernández, Jorge Pedraza García, Paula Pedraza Ramírez, Francisco Javier Montero-Pérez, Carmen Lucena Aguilera, Francisco de Borja Quero Espinosa, Ángela Cobos Requena, Esperanza Muñoz Triano, Inmaculada Bajo Fernández, María Calderón Caro, Sierra Bretones Baena, Iria Miguens Blanco, Ioana Muñoz Betegón, Dariela Edith Micheloud Giménez, Jorge Sánchez-Tembleque Sanchez, Belén Macías Bou, Paloma Díez Romero, María Fernández Cardona, Leonor Andrés Berián, Lucía González Ferreira, Rocio Hernando González, María Eugenia Rodríguez Palma, Javier Varona Castrillo, Daniel Aguilar Pérez, Alberto Álvarez Madrigal, Marta Iglesias Vela, Mónica Santos Orús, Rudiger Carlos Chávez Flores, Miguel Moreno Martín, Albert Carbó Jordá, Enrique González Revuelta, Sara Sánchez Aroca, Rafael Antonio Pérez-Costa, María Rodríguez Romero, Esperanza Marín Arranz, Elena Ramírez Gallardo, Ana Palací Bataller, Paula Sánchez Herrero, Julia Martínez-Ibarreta Zorita, Irene Cabrera Rodrigo, Beatriz Mañero Criado, Raquel Torres Gárate, Rebeca González González, Eva Quero Motto, Nuria Tomas Garcia, Laura Bernal Martínez, Marina Carrión Fernández, Carmen Escudero Sánchez, Belén Morales Franco, Maria Adroher Muñoz, Ester Soy Ferrer, Eduard Anton Poch Ferrer, Jeong-Uh Hong Cho, Patricia Trent Español, Fernando López López, Jorge Navarro Calzada, Belén Gros Bañeres, Cristina Martín Durán, María Teresa Escolar Martínez-Berganza, Iciar González Salvatierra, Sara Gayoso Martín, María Goretti Sánchez Sindín, María Esther Fernández Álvarez, Martina Silva Penas, Margarita Puiggali Ballard, Maria Carmen Petrus Rivas, Bárbara Gómez Gómez, Isabel Cirera Lorenzo, Aitor Alquezar Arbé, Isel Borrego Yanes, Adriana Laura Doi Grande, Sergio Herrera Mateo, Olga Trejo Gutiérrez, Paola Ponte Márquez, Carlos Romero Carrete, Sayoa Francesena González, Celia Rodríguez Valles, Verónica Vázquez Rey, Antonio Rodríguez Mejía, Elena Carrasco Fernández, Mónica Cañete, Mar Sousa, Laura Molina, Esther Ruescas, Pedro Ruiz Asensio, María Martínez Juan, Beatriz Paderne Díaz, Eva De las Nieves Rodríguez, Gema Gómez García, Amparo Fernández-Simón Almela, Esther Pérez García, Pedro Rivas Del Valle, María Sánchez Moreno, Rafaela Rios Gallardo, Laura Redondo Lora, Ana Gómez Caminero, Claudio Bueno Mariscal, María Amparo Berenguer Diez, María Ángeles De Juan Gómez, María Luisa López Grima, Rigoberto Jesús Del Rio Navarro, Pere Rull Bertrán, Marta Masid Barcon, Fiorella Granado Fronzo, Núria Perelló Viola, Lourdes Hernández-Castells, José Bermejo Noceda, María Teresa Sánchez Moreno, Raquel Benavent Campos, Alejandro Cortés Soler, María Teresa Maza Vera, Raquel Rodríguez Calveiro, Paz Balado Dacosta, Violeta Delgado Sardina, Emma González Nespereira, Elena Sánchez Fernández-Linares, Ángel García García, Manuel Ángel Palomero Martín, Jesús Ángel Sánchez Serrano, María Jesús Soriano Pérez, José Ramón Oliva Ramos, Virginia Carbajosa Rodríguez, Susana Sánchez Ramón, Maria José Marchena, Jose Maria Santos Martin, Jesús Santianes Patiño, Octavio Gutiérrez Alcalá, Pablo Herrero Puente, Alba Martínez Alonso, Ángela López Carrillo, Belén Pérez Fernández, Carmen Pérez Fonseca

**Affiliations:** 1 Emergency Department, Hospital Clínic, IDIBAPS, Barcelona, Catalonia Spain; 2 Department of Medicine, Faculty of Medicine, University of Barcelona, Barcelona, Catalonia Spain; 3 Emergency Department, Hospital de La Santa Creu I Sant Pau, Barcelona, Catalonia Spain; 4 Emergency Department, Hospital Clínico San Carlos, IDISSC, Complutense University, Madrid, Spain; 5 Emergency Department, Hospital Universitario de Canarias, University of La Laguna, San Cristóbal de La Laguna, Canary Islands, Tenerife, Spain; 6 Emergency Department, Hospital Universitari de Bellvitge, L’Hospitalet de Llobregat, Barcelona, Catalonia Spain; 7 Emergency Department, Hospital Universitario Dr. Balmis, Alicante, Spain; 8 Emergency Department, Hospital Universitari de Vic, Barcelona, Catalonia Spain; 9 Emergency Department, Hospital del Nalón, Langreo, Asturias Spain; 10 Emergency Department, Hospital Altagracia, Manzanares, Ciudad Real Spain; 11 Emergency Department, Hospital Nuestra Señora del Prado de Talavera de La Reina, Toledo, Spain; 12 Emergency Department, Hospital Universitario Vinalopó, Elche, Alicante Spain; 13 Emergency Department, Hospital de Móstoles, Madrid, Spain; 14 Emergency Department, Hospital Virgen del Rocio, Seville, Spain; 15 Emergency Department, Hospital General Universitario Dr. Peset, Valencia, Spain; 16 Emergency Department, Hospital Universitario Son Espases, Palma, Spain; 17 Emergency Department, Clinica Universitaria Navarra de Madrid, Madrid, Spain; 18 Emergency Department, Hospital Clinico Universitario de Valencia, Valencia, Spain; 19 Emergency Department, Hospital Alvaro Cunqueiro, Vigo, Pontevedra, Spain; 20 Emergency Department, Hospital Universitario de Salamanca, Salamanca, Spain; 21 Emergency Department, Hospital de Zumárraga, Guipúzcoa, Spain; 22 Emergency Department, Hospital Virxe da Xunqueira, Cee, A Coruña, Spain; 23 Emergency Department, Hospital Universitario Los Arcos del Mar Menor, San Javier, Murcia, Spain; 24 Emergency Department, Hospital Universitario Río Ortega, Valladolid, Spain; 25 Emergency Department, Hospital Juan Ramón Jiménez, Huelva, Spain; 26 Emergency Department, Hospital Central de Asturias, Oviedo, Spain

**Keywords:** Delirium, Neuroleptics, Benzodiazepines, Emergency department, Mortality, Outcome

## Abstract

To investigate factors related to the development of hyperactive delirium in patients during emergency department (ED) stay and the association with short-term outcomes. A secondary analysis of the EDEN (Emergency Department and Elderly Needs) multipurpose multicenter cohort was performed. Patients older than 65 years arriving to the ED in a calm state and who developed confusion and/or psychomotor agitation requiring intravenous/intramuscular treatment during their stay in ED were assigned to delirium group. Patients with psychiatric and epileptic disorders and intracranial hemorrhage were excluded. Thirty-four variables were compared in both groups and outcomes were adjusted for age, sex, Charlson Comorbidity Index, Barthel Index and polypharmacy. Hyperactive delirium that needed treatment were developed in 301 out of 18,730 patients (1.6%). Delirium was directly associated with previous episodes of delirium (OR: 2.44, 95% CI 1.24–4.82), transfer to the ED observation unit (1.62, 1.23–2.15), chronic treatment with opiates (1.51, 1.09–2.09) and length of ED stay longer than 12 h (1.41, 1.02–1.97) and was indirectly associated with chronic kidney disease (0.60, 0.37–0.97). The 30-day all-cause mortality was 4.0% in delirium group and 2.9% in non-delirium group (OR: 1.52, 95% CI 0.83–2.78), need for hospitalization 25.6% and 25% (1.09, 0.83–1.43), in-hospital mortality 16.4% and 7.3% (2.32, 1.24–4.35), prolonged hospitalization 54.5% and 48.6% (1.27, 0.80–2.00), respectively, and 90-day post-discharge combined adverse event 36.4% and 35.8%, respectively (1.06, 0.82–2.00). Patients with previous episodes of delirium, treatment with opioids and longer stay in ED more frequently develop delirium during ED stay and preventive measures should be taken to minimize the incidence. Delirium is associated with in-hospital mortality during the index event.

## Introduction

Delirium is an acute and often fluctuating disturbance in attention and awareness, that is frequently associated with psychomotor hyperactivity and is extremely common among older hospitalized adults. Its incidence has been estimated at 29% to 64% in general medical wards, 50% after high-risk surgical procedures, and up to 75% in patients receiving mechanical ventilation in the intensive care unit [1–3]. Development of delirium during hospitalization is associated with adverse outcomes, including an increased risk of falls, functional decline, dementia, prolonged hospitalization, institutionalization, and death [4].

The development of delirium in the emergency department (ED) is less frequently reported, probably because the time a patient stays in the ED (usually hours) is shorter than that spent in hospitalization (days). In parallel, there are only a few studies on delirium from the ED perspective. In a very recent systematic review of delirium including 315 studies, the authors only identified 7 studies performed in the ED [5]. Among the circumstances limiting information of older patients developing delirium in the ED, the scarcity of prospective, large, unselected multicenter cohorts specifically recruiting this population in EDs is of note. Recent efforts have been made in this regard. Two examples are the EGERS cohort including the participation of 36 EDs located in 9 European countries that recruited 5,767 patients aged 65 or older [6], and the EDEN cohort including 52 Spanish EDs that recruited 25,557 patients within the same age-frame [7, 8]. We used this latter cohort to investigate factors associated with the development of delirium in the ED and its potential relationship with short-term outcomes.

## Methods

### SIESTA network and the EDEN project

The SIESTA (Spanish Investigators in Emergency Situations TeAm) research network was created in 2020. It is made up of researchers who mainly work in the ED and its main purpose is to manage multidisciplinary research challenges in real clinical practice from a multicentric perspective and with a wide representation of Spanish EDs. The network has a stable coordinating core, and researchers from individual EDs can join when a research challenge arises according to their interest and availability [9, 10].

The EDEN (Emergency Department and Elderly Needs) challenge was launched by the SIESTA network with the primary objective of increasing knowledge about socio-demographic, organizational, baseline, clinical care and outcomes of the population ≥ 65 years consulting in Spanish EDs. To this end, a retrospective multipurpose registry was designed. The EDEN cohort included all patients who consulted in 52 Spanish EDs (17% of the EDs of the Spanish public network with a catchment area of about 25% of the Spanish population) between April 1 and 7, 2019 (7 days). There was no reason for exclusion, and the EDs included all patients seen during the study period regardless of the reason for consultation. Extensive details of the EDEN registry have been published in detail previously [8, 11, 12].

### EDEN-27 study design

The EDEN-27 study was specifically designed to analyze the factors associated with the development of hyperactive delirium in older patients while they are attended in the ED. We included all the patients of the EDEN registry that arrived at the ED in a calm state (with no confusion, decreased level of consciousness or agitation) in whom treatments administered during ED stay were recorded. Patients requiring intravenous/intramuscular neuroleptics or benzodiazepines because of psychomotor hyperactivity or confusion were included in the group of delirium. The use of these drugs per via oral was not considered due to the difficulty in distinguishing when they were administered as part of the regular treatment of the patient. The remaining patients formed the control group (non-delirium). For patients developing delirium, we recorded the 10 main groups of ED diagnosis (for all patients and for hospitalized patients) as well as the drugs used to treat delirium. In addition, we also recorded the percentage of patients in the non-delirium groups with these 10 diagnoses.

As independent variables, we recorded 2 socio-demographic variables (age, sex), the Charlson Comorbidity Index (CCI) and 10 comorbidities (hypertension, diabetes mellitus, coronary artery disease, cerebrovascular disease, chronic heart failure, chronic kidney disease, chronic pneumopathy, neoplasia, chronic liver disease, dementia), functional capacity assessed with the Barthel Index (BI) and 5 other variables referring to baseline status (ability to walk, previous episodes of delirium, previous diagnosis of depression and cognitive impairment, fall during previous 6 months), number of chronic medications and 11 individual groups of drugs (rein-angiotensin system inhibitors, diuretics, benzodiazepines, oral anti-diabetics, insulin, beta-blockers, anti-depressants, opioids, non-steroidal anti-inflammatory drugs, neuroleptics, oral corticosteroids, anti-epileptics), and 2 variables related to ED stay (patient transfer to observation area, total time of patient stay in the ED).

### Outcomes

The outcomes assessed consisted of 30-day-all-cause mortality and need for hospitalization (for all patients), in-hospital mortality and hospitalization longer than 7 days (for patients that were hospitalized), and post-discharge combined adverse event (ED revisit, hospitalization or death, whatever the cause) occurring during the 90 days following discharge (for patients discharged alive, i.e., patients dying during the index episode were not taken into account for this outcome). Outcomes were checked by review of all the patient health care records, including primary care records and national registry of deaths, and were adjudicated at the local level by the principal investigator of each center without external review.

### Statistical analysis

Quantitative variables are expressed as median and interquartile range (IQR), and qualitative variables as the number of cases and percentages. For comparisons, we used the Kruskal–Wallis test and the Chi-square test, respectively. The factors associated with the development of delirium in the ED were investigated by logistic regression. We created a multivariate model including all the independent variables included in the present study (enter function). Continuous variables were dichotomized as follows: age ≥ 85 years, severe or absolute functional dependence (BI < 60 points), severe comorbidity (CCI > 4 points) and severe polypharmacy (> 9 chronic drugs). The relationship between delirium with outcomes was investigated using logistic regression, first unadjusted, and then in an adjusted model that included 5 covariates defined a priori as we considered they could be associated with outcomes: age, sex, CCI, BI and number of chronic drugs.

For all comparisons, statistical significance was accepted if the *p* value was < 0.05 or if the 95% confidence interval (CI) of the risk estimations excluded the value 1. All the analyses were performed with the SPSS package, version 24 (IBM, Armonk, New York, USA). Figures were produced using Excel and Power Point 2016 (Microsoft Corporate Office, Redmond, Washington, USA).

### Ethics

The EDEN project was approved by the Clinical Research Ethics Committee of the Hospital Clínico San Carlos de Madrid (protocol HCSC/22/005-E). Due to the non-interventional design of the registry, Spanish legislation allows central Ethical Committee approval accompanied by notification to the local Ethical Committees. Due to the retrospective and non-interventional design of the EDEN project, patient informed consent was waived. The present study was carried out in strict compliance with the principles of the Declaration of Helsinki.

## Results

**Fig. 1:**
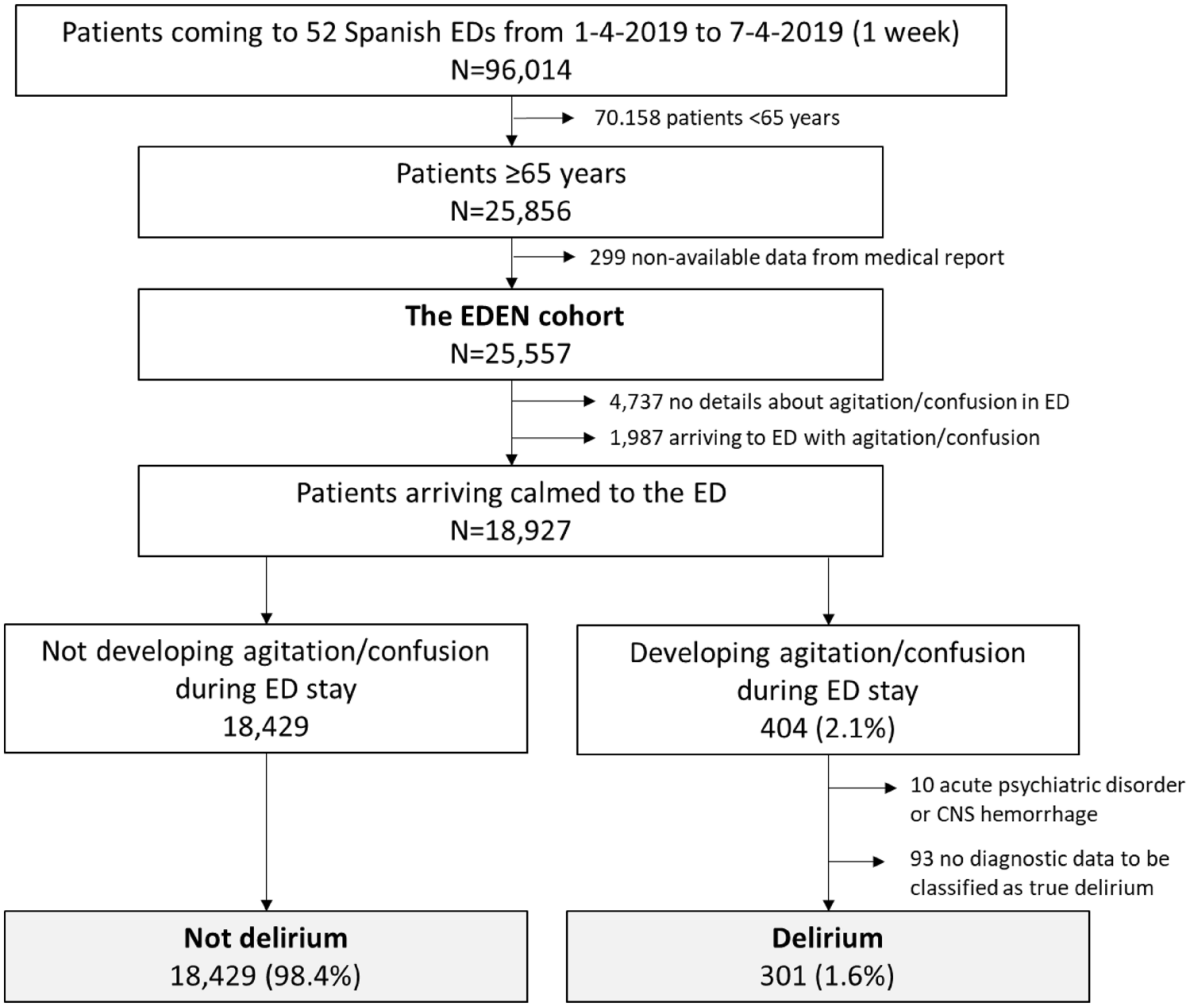
Flow chart for patient inclusion in the EDEN-27 study. *ED* emergency department

**Fig. 2:**
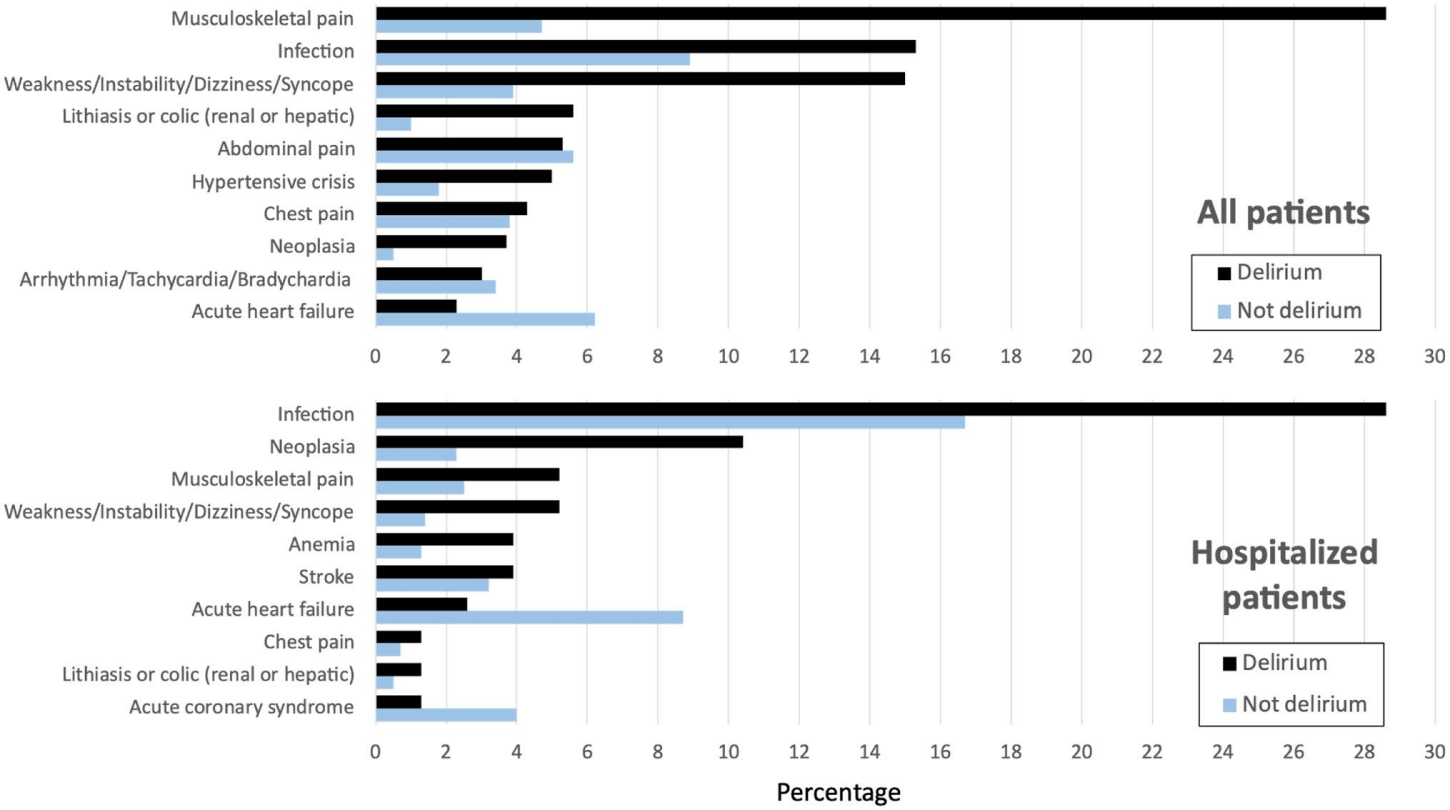
Bar graph with the 10 most frequent causes of emergency department consultation in patients developing delirium during emergency department stay with comparison of frequency in the population that did not develop delirium. Upper graph corresponds to all patients and bottom graph to patients that were hospitalized

We included 18,730 older patients in the EDEN-27 study, and 301 (1.6%) were considered to have developed delirium during their stay in the ED (Fig. 1). The median length of ED stay for the whole cohort was 3:44 h (IQR: 2:00–6:47; with 5.8% of patients staying longer than 24 h), and patients developing delirium staying longer (median: 4:33, IQR: 2:45–8:55) than the rest (median: 3:42, IQR: 2:00–6:47; *p* < 0.001). The most frequent diagnoses of patients with delirium were musculoskeletal pain (28.6%), infection (15.3%) and weakness, instability, dizziness or syncope (15.0%) (Table 1). Benzodiazepines were used in around two-thirds of the patients and neuroleptics in one-third, while 7 patients (2.3%) required whole-body physical retainment. Among the 10 most frequent diagnoses in the delirium group, 5 were clearly more frequent compared to the non-delirium group (musculoskeletal pain, infection, weakness/instability/dizziness/syncope, lithiasis or colic, neoplasia), while one was less frequent (acute heart failure). Although the frequency of the main 10 diagnoses changed when analyzing hospitalized patients with delirium, differences with respect to frequency in patients without delirium were very similar to those observed in the analysis of all patients (with the addition of acute coronary syndrome, which was more frequent in the non-delirium group) (Fig. 2).

**Table 1:** Main diagnoses of patients developing delirium in the emergency department included in the EDEN-27 study

	Patients with delirium *N* = 301 *n* (%)
Main diagnosis in the ED*	
Musculoskeletal pain	**86 (28.6)**
Axial pain	58 (19.3)
Headache	8 (2.7)
Arms and limbs	16 (5.3)
Post-trauma	4 (1.3)
Infection	**46 (15.3)**
Respiratory tract	19
Urinary tract	12
Gastroenteritis	5
Other	10
Weakness/instability/dizziness/syncope	**45 (15.0)**
Lithiasis or colic (renal or hepatic)	**17 (5.6)**
Abdominal pain	**16 (5.3)**
Hypertensive crisis	**15 (5.0)**
Chest pain	**13 (4.3)**
Neoplasia	**11 (3.7)**
Arrhythmia/tachycardia/bradycardia	**9 (3.0)**
Acute heart failure	**7 (2.3)**
Other diagnosis individually considered	**72 (24.0)**
Drugs used to treat delirium*	
Benzodiazepines	**205 (68.1)**
Intravenous	137 (45.5)
Intramuscular	51 (16.9)
Oral plus parenteral	21 (6.9)
Neuroleptics	**106 (35.2)**
Intravenous	67 (22.3)
Intramuscular	33 (11.0)
Oral plus parenteral	6 (2.0)
Complete physical retainment	**7 (2.3)**

*ED* emergency department

*Some patients simultaneously received more than one diagnosis, or benzodiazepines and neuroleptics were used in combination and through more than one route; accordingly, the summatory surpasses 100%

**Fig. 3:**
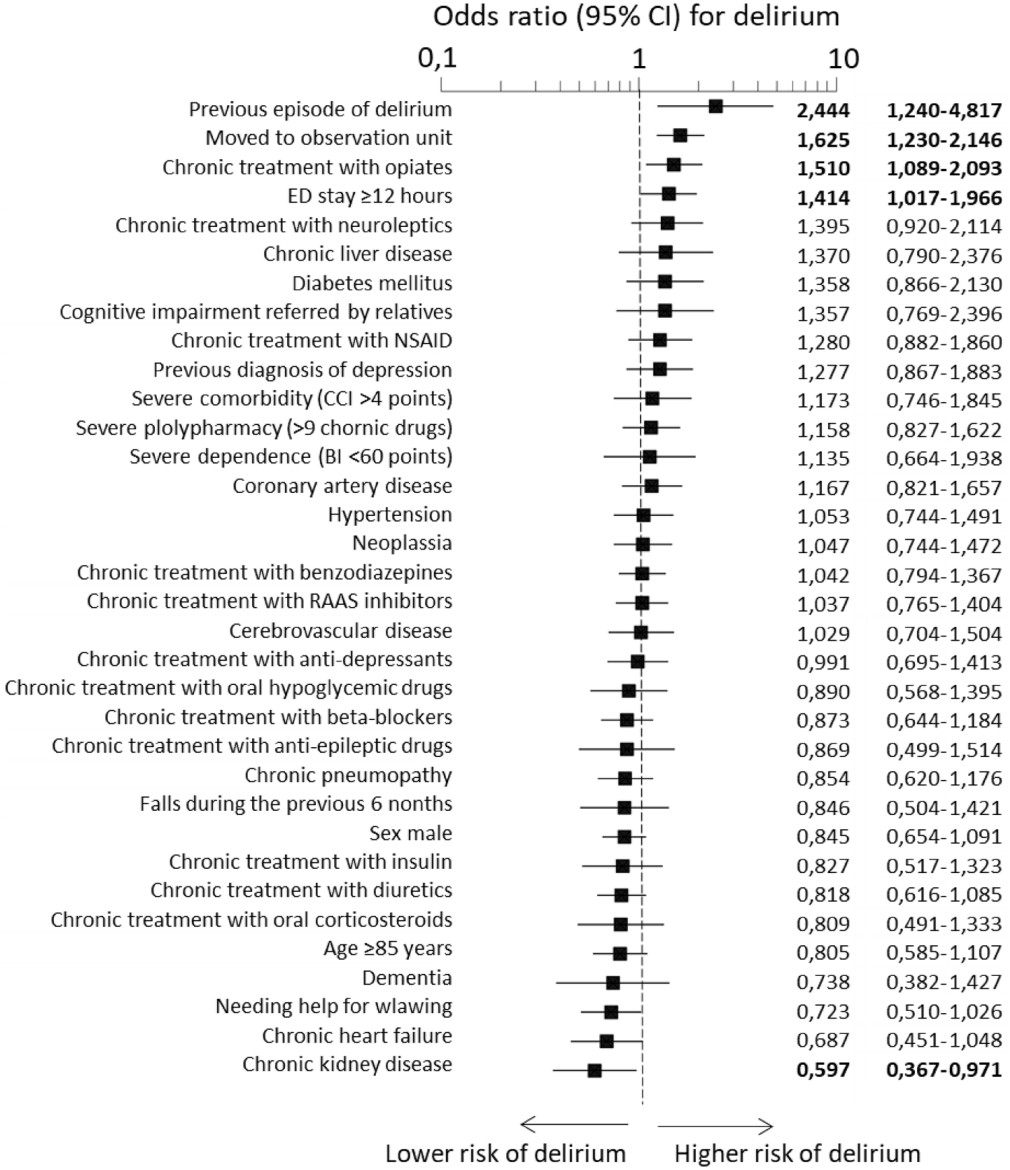
Adjusted associations for variables analyzed in the EDEN-27 study with the development of delirium during patient stay in the emergency department (adjustment was performed by forcing the entrance of all variables in the multivariate model). Odds ratio in bold numbers denote statistically significant differences (*p* < 0.05). *OR* odds ratio, *CI* confidence, *NSAID* non-steroidal anti-inflammatory drug, *ED* emergency department, *RAAS* renin–angiotensin–aldosterone system, *CCI* Charlson comorbidity Index, *BI* Barthel index.

Patients with delirium differed from the rest in 10 out of the 34 independent variables (Table 2). The adjusted model showed that the development of delirium in the ED was directly associated with previous episodes of delirium (odds ratio [OR]: 2.44, 95% CI 1.24–4.82), being moved to the ED observation unit (OR: 1.62, 95% CI 1.23–2.15), chronic treatment with opiates (OR: 1.51, 95% CI 1.09–2.09) and ED length of stay longer than 12 h (OR: 1.41, 95% CI 1.02–1.97), and was indirectly associated with chronic kidney disease (CKD) (OR: 0.60, 95% CI 0.37–0.97) (Fig. 3).

**Table 2:** Characteristics of the patients developing delirium in the emergency department included in the EDEN-27 study and comparison with those that did not develop delirium

	Delirium *N* = 301 *n* (%)	No delirium *N* = 18,429 *n* (%)	*P* value
Sociodemographic			
Age (in years) (median (IQR))	76 (71–83)	78 (71–84)	**0.01**
Female sex	170 (57.8)	9,715 (53.9)	0.18
Comorbidities			
Comorbidity (points, by the Charlson Comorbidity Index) (median (IQR))	2 (0–3)	2 (0–3)	0.75
Hypertension	215 (71.4)	13,288 (72.1)	0.80
Diabetes mellitus	95 (31.6)	5,298 (28.7)	0.28
Coronary artery disease	50 (16.6)	3,055 (16.6)	0.99
Cerebrovascular disease	34 (11.3)	2,081 (11.3)	1.00
Chronic heart failure	34 (11.3)	2,843 (15.4)	**0.04**
Chronic kidney disease	23 (7.6)	2,182 (11.8)	**0.03**
Chronic pneumopathy	53 (17.6)	3,773 (20.5)	0.22
Neoplasia	63 (20.9)	3,488 (18.9)	0.38
Chronic liver disease	17 (5.6)	673 (3.7)	0.07
Dementia	23 (7.6)	1,323 (7.2)	0.76
Baseline status			
Functionally dependent (Barthel Index < 100 points)	89 (29.5)	6,773 (30.8)	0.65
Needing help for walking or unable to walk	68 (22.6)	4,778 (26.0)	0.19
Previous episodes of delirium	11 (3.7)	258 (1.4)	**0.001**
Previous diagnosis of depression	51 (16.9)	2,304 (12.5)	**0.02**
Previous diagnosis of cognitive impairment	34 (11.3)	1,727 (9.4)	0.26
Having had falls in the previous 6 months	17 (5.6)	1,124 (6.1)	0.74
Chronic treatments			
Number of chronic medications (median (IQR))	6 (4–10)	6 (3–9)	0.37
Renin–angiotensin system inhibitors	166 (55.1)	10,167 (55.2)	0.99
Diuretics	107 (35.5)	7,447 (40.4)	0.09
Benzodiazepines	106 (35.2)	5,631 (30.6)	0.08
Oral anti-diabetics	76 (25.2)	4,189 (22.7)	0.30
Beta-blockers	73 (24.3)	5,037 (27.3)	0.23
Anti-depressants	73 (24.3)	3,706 (20.1)	0.08
Opiates	60 (19.9)	2.387 (13.0)	** < 0.001**
Non-steroidal anti-inflammatory drugs	39 (13.0)	1,621 (8.8)	**0.01**
Neuroleptics	34 (11.3)	1,373 (7.5)	**0.01**
Insulin	28 (9.3)	1,750 (9.5)	0.91
Oral corticosteroids	19 (6.3)	1,314 (7.1)	0.58
Anti-epileptic	15 (5.0)	831 (4.5)	0.69
Factors related to ED stay			
Transferred to an ED observation unit	119 (39.5)	5,117 (27.8)	** < 0.001**
Time in the ED (until discharge or hospitalization) (in hours) (median (IQR))	4:33 (2:45–8:52)	3:42 (2:00–6:46)	** < 0.001**

*ED* emergency department, *IQR* interquartile range

*P* values in bold numbers denote statistically significant differences (p < 0.05)

Considering the whole cohort, a total of 537 patients died during the following 30 days (2.9%; 204 excluded because the length of follow-up was < 30 days—1.1%—) and 4,688 required hospitalization (25.0%). Among the hospitalized patients, 2,227 had prolonged hospitalization (48.8%, 123 excluded due to no data available on length of hospitalization—2.6%—) and 351 (7.5%) died during hospitalization. Among 18,335 patients discharged alive, 16,347 were followed for 90 days (in 1,988, 10.8%, follow-up was unavailable or they were followed < 90 days) and a post-discharge combined event was present in 5,898 patients (36.1%).

The 30-day all-cause mortality was 4.0% in the delirium group and 2.9% in the non-delirium group. The adjusted OR in patients developing delirium was 1.52 (95% CI 0.83–2.78). For the remaining outcomes, the need for hospitalization was present in 25.6% and 25.0% of patients, respectively (OR: 1.09, 95% CI 0.83–1.43), the in-hospital mortality for hospitalized patients was 16.4% and 7.3%, respectively (OR: 2.32, 95% CI 1.24–4.35), prolonged hospitalization was 54.5% and 48.6%, respectively (OR: 1.27, 95% CI 0.80–2.00), and the 90-day post-discharge combined adverse event for patients discharged alive after the index event was 36.4% and 35.8%, respectively (OR: 1.06, 95% CI 0.82–2.00) (Table 3).

**Table 3:** Unadjusted and adjusted associations between the development of delirium and short-term outcomes

	Delirium *N* = 301 *n* (%)	No delirium *N* = 18,429 *n* (%)	Unadjusted OR (95% CI)	Adjusted* OR (95% CI)
30-day all-cause mortality (all patients)	12 (4.0)	525 (2.9)	1.41 (0.79–2.54)	1.52 (0.83–2.78)
Need for hospitalization (all patients)	77 (25.6)	4,611 (25.0)	1.03 (0.79–1.34)	1.09 (0.83–1.43)
In-hospital mortality (in hospitalized patients)	13 (16.9)	338 (7.3)	**2.57 (1.40–4.71)**	**2.32 (1.24–4.35)**
Prolonged hospitalization (> 7 days) (in hospitalized patients)	42 (54.5)	2,180 (48.6)	1.27 (0.81–1.99)	1.27 (0.80–2.00)
90-day post-discharge combined adverse event (in patients discharged alive)	95 (36.4)	5,733 (35.8)	1.02 (0.79–1.32)	1.06 (0.82–1.37)

Bold odds ratio denotes statistical significance (*p* < 0.05)

*OR* odds ratio, *CI* confidence interval

*Covariates included in the adjusted model were the following: age, sex, Charlson Comorbidity Index, Barthel Index and number of chronic drugs

## Discussion

EDEN-27 is an exploratory study that investigated the factors related to the development of delirium in older patients while they are attended in the ED and also analyzed its impact on short-term outcomes. The main novelty of this study is that it is exclusively focused on the ED, using a large cohort of unselected consecutive patients, while most previous studies have looked at the development of delirium during or including the hospitalization period, even if patients had been selected in the ED, and used shorter cohorts. Four main findings merit highlighting. First, less than 2% of older patients develop symptoms of delirium needing pharmacological treatment while they are in the ED. Second, previous episodes of hyperactive delirium and treatment with opiates were the two patient-dependent factors associated with an increased risk of delirium development, while CKD was associated with a decreased risk. Third, moving patients to an ED observation unit as well as prolonged stay in the ED were the two environment-related factors associated with increased risk of delirium. Fourth, the development of delirium in the ED increases the risk of death in patients requiring hospitalization during the index event (in-hospital mortality), while the trend to an increase in 30-day mortality found in the present study for the whole cohort did not achieve statistical significance.

The low incidence of delirium in the ED is probably related to the short time that patients stay in the ED (only 6% stayed longer than 24 h) and that we only considered hyperactive delirium and only if it was developed during ED stay but not during hospitalization. The few studies performed in EDs have shown a higher percentage of incidence (between 9 and 16%), although they included a lower number of non-consecutive patients and included patients in whom delirium developed during the first hours or days of hospitalization. A multicenter Canadian cohort of 612 patients aged ≥ 65 years with an ED length of stay ≥ 8 h and hospitalized showed an incidence of delirium of 11–12% when followed to 24 h after hospitalization [13–15], with delirium starting within a mean delay of 47 h after ED admission [14]. Similarly, an Italian cohort of 330 individuals aged ≥ 75 years without delirium coma, aphasia, stroke, language barrier, psychiatric disorder or alcohol abuse at ED entry reported 16% of delirium during the 3 days following hospitalization [16]. A study in the United States reported that 9% of 695 ED comers aged ≥ 65 years developed delirium [17]. Finally, a German study screened 133 consecutive ED patients aged ≥ 75 years for delirium and found an incidence of 14% [18]. Interestingly, they evidenced that prospective use of the Confusion Assessment Method (CAM) allowed the diagnosis of up to two-thirds of the patients that were unsuspected (and undiagnosed) by emergency physicians. Therefore, the 2% of incident delirium strictly developed in the ED and diagnosed by clinical criteria found in the EDEN-27 study probably matches these higher incidences reported in the aforementioned cohorts in which longer follow-up periods and more sensitive tools for delirium detection were used.

The systematic review of 331 studies (only 7 run in EDs) by Ormseth et al*.* including 101,144 patients (24 015 with delirium) identified 33 predisposing and 112 precipitating factors associated with delirium [5]. A previous episode of delirium was one factor and, accordingly, it must be mandatory to record this item during the clinical interview, even at the first triage contact [19], as early identification of this risk factor can be followed by preventive measures. We also identified chronic treatment with opiates as a risk factor in our cohort. Narcotic analgesics were found to be a predisposing factor of delirium in one study and psychoactive medication (unspecified) in 3. The role of chronic medication, however, is not clear, as small studies identified chronic use of opioids (for analgesia, to reduce pain, in comparison with patient-controlled analgesia) as a protective factor [20, 21]. On the other hand, opiate use was found to be the most frequent drug precipitating delirium. It was present in 14 studies, while benzodiazepines were involved in 11 studies, neuroleptics in 5 and psychoactive drugs in 2. When looking at the main diagnoses of patients developing delirium (Fig. 2), it can also be seen that certain diagnoses are clearly more frequent compared to those of patients without delirium, as in the case of musculoskeletal pain, suggesting that such entities (along with their treatments) could also be linked with the development of delirium in the ED. Future studies specifically designed to address this hypothesis are needed to definitively confirm our findings.

By contrast, we found that CKD was inversely related to delirium development. Interestingly, the above-mentioned systematic review only identified one study linking CKD with increased risk of delirium development, while a neutral effect has been found in several other studies [5]. On the other hand, while advanced age was identified as being related to delirium in most studies, we failed to demonstrate a significant relationship in our study. Several previous studies have included patients with a wide range of age, favoring an increased risk of delirium in the elderly compared to young populations; conversely, as we only included patients aged 65 years and more, this relationship might not have been as clear in the EDEN-27 study as in other studies. In fact, the same finding of a neutral effect of age on delirium when cohorts exclusively formed by older patients are evaluated has previously been reported [13].

ED-related factors favoring the development of delirium have been poorly explored. We found that moving patients within the ED and prolonged ED stays are associated with a higher frequency of delirium. Interestingly, a longer length of ED stay was associated with a higher incident delirium in Canadian and Italian cohorts [13, 16], even after adjusting for age and cognitive impairment [16]. As is often the case in observational studies, it is not possible to distinguish between association and causation. In this sense, patients with delirium stay longer in the ED because of organizational problems, because they are more critical or frail or as a consequence of delirium. For this reason, although any measure making ED stay as short as possible in older patients (as well as limiting changes of patient placement) would probably help to reduce, to some extent, the risk of the development of delirium in the ED, these interventions should be evaluated in prospective (and, if possible, randomized) studies. In the same regard, the absence of patient mobilization in the ED due to bladder catheters, intravenous fluid therapy or physical restraint is also associated with the development of delirium [13] and should also be avoided. The implementation of specific changes in structural design and age-adapted protocols should be firmly recommended in every ED [22, 23].

In the EDEN-27 study, there was a marked (adjusted OR of 2.32) and significant increase of in-hospital mortality for admitted patients with delirium. Although the 30-day mortality of the whole cohort was also increased (adjusted OR of 1.52), it did not reach statistical significance. The interpretation of this apparent incongruence should be made with caution. Hypothetically, it could be due to the fact that hospitalized patients with delirium were sicker than those without delirium, while discharged patients were healthier. Evaluation of the causes leading to hospitalization in both groups (Fig. 2) does not clarify whether this was the case. On the other hand, as we did not collect the specific causes of death, this explanation for the mild difference in in-hospital and 30-day mortality in patients with delirium remains entirely speculative. A number of other adverse outcomes had previously been reported in ED patients developing delirium in the US cohort [17]: longer median lengths of stay (4 vs. 2 days), greater likelihood of requiring intensive care unit admission (13% vs. 6%), need for a long-term care facility at discharge (37% vs. 9%), and higher 30-day mortality (6% vs. 1%) and 30-day readmission (27% vs 13%). Nonetheless, and aside from the increased in-hospital mortality, we failed to demonstrate any significant increase in any other outcome in patients developing delirium.

### Limitations

First, the 52 participating EDs were not chosen at random but rather expressed their interest in participating. However, the broad representation, both territorially (14 of the 17 autonomous communities were represented) and in terms of typology (including university, high-technology and regional hospitals), means that the bias in this regard is likely small. Second, the analyses were not carried out by nosology groups but rather globally. This may mean that the findings are conditioned by certain processes. Nonetheless, the EDEN-27 study is exploratory in nature and it is designed to capture all the spectrum of ED comers and is not limited to a single disease or group of diseases. Third, this was a secondary analysis of a multipurpose cohort, and thus, the associations presented may be influenced by factors not covered in the cohort design, and it was not possible to clarify if the associations are a cause or a consequence. Frailty, in particular, is a key aspect in older patients, and although we recorded previous falls as a subrogate, complete assessment of frailty was lacking [24–26]. Therefore, the findings are exclusively hypothesis-generating and should be confirmed by studies specifically designed for this purpose. Fourth, diagnosis of delirium was not based on the CAM but rather on clinical findings. Certainly, this limited the inclusion in the EDEN-27 study to the most symptomatic patients, as it is well known that use of the CAM increases the detection of delirium [18] and that hypoactive forms of delirium are more difficult to detect than the hyperactive forms when diagnosis is based on clinical grounds. Fifth, we did not record the time from ED arrival to the development of delirium, the percentage of patients developing delirium during hospitalization or the number of patients treated with oral neuroleptics/benzodiazepines (which guidelines describe as a first-line treatment of delirium). Knowing this important information could have helped to better interpret the results. Sixth, the inclusion of patients in the EDEN cohort was done by episodes rather than by patients. However, given that the inclusion period was very short (7 days), the likelihood of a repeat visit by a particular patient can be considered low. Finally, the EDEN-27 study only included Spanish EDs, and external validation in other countries is needed, as differences in health care systems in general, and ED organization in particular, are wide worldwide and can influence the rate of delirium development in the ED.

## Conclusion

Some patient- and ED-related factors are associated with the risk of development of hyperactive delirium in the ED. For patients needing hospitalization, delirium is associated with a higher in-hospital mortality. Older patients with known risk factors, especially previous episodes of delirium, should be routinely assessed and strategies to minimize the risk of delirium development should be quickly implemented. Among these, a reduction in patient stay in the ED and avoidance of patient movement within the ED could contribute to decreasing incident delirium in EDs.

## Data Availability

The datasets used and analyzed during the curing study are available from the corresponding author on reasonable request.
